# A systematic review of barriers and motivators to physical activity in elderly adults in Iran and worldwide

**DOI:** 10.4178/epih.e2019049

**Published:** 2019-11-29

**Authors:** Soudabeh Yarmohammadi, Hossein Mozafar Saadati, Mohtasham Ghaffari, Ali Ramezankhani

**Affiliations:** 1 Student Research Committee, Department and Faculty of Health Education and Health Promotion, Shahid Beheshti University of Medical Sciences, Tehran, Iran; 2 Department of Epidemiology, School of Public Health and Safety, Shahid Beheshti University of Medical Sciences, Tehran, Iran; 3 Environmental and Occupational Hazards Control Research Center, School of Public Health and Safety, Shahid Beheshti University of Medical Sciences, Tehran, Iran; 4 Department of Public Health, School of Public Health and Safety, Shahid Beheshti University of Medical Sciences, Tehran, Iran

**Keywords:** Motivation, Sedentary behavior, Exercise, Motor activity, Aging

## Abstract

**OBJECTIVES:**

This study was conducted to identify and characterize the barriers and motivations to physical activity (PA) for elderly adults in Iran and other countries.

**METHODS:**

We searched 6 databases (PubMed, Embase, Scopus, Web of Science, Magiran, and the Scientific Information Database) from 2000 to the November 2017, using “aged 60 and over,” “physical activity” or “exercise,” and “motivator” and “barrier” as keywords. Two reviewers independently performed the search, screening, and quality assessment of the studies.

**RESULTS:**

In total, 34 papers were finally included in the study. The most important barriers, based on the frequency of factors, included physical problems, having no companions, and physical barriers to walking. The motivators included improving one’s physical condition, being social, and suitability of the physical environment.

**CONCLUSIONS:**

Important motivators and barriers to PA were more closely related to intrapersonal factors than to the interpersonal and environmental domains. The barriers and motivators to PA in the elderly were not markedly different between Iran and other countries. Therefore, a general strategy could be designed to improve PA in the elderly.

## INTRODUCTION

On one hand, physical activity (PA) helps elderly people to improve their strength and flexibility, although the most effective type of activity is still unclear [1]. On the other hand, the elderly—due to health problems and insufficient facilities—are more likely to face barriers to access than others [2].

For adults aged 60 and over, important motivations for PA have been reported to include social support, health benefits, and enjoyment, while the main barriers have been found to be insufficient guidance and a lack of role models [3]. For people aged over 80 years, the most important motivators and barriers have been reported to be the health benefits of PA, various types of fears, individual preferences, and social support [4]. In general, due to the increasing percentage of the older population in developing (low-and middle-income) countries and the burden of health care and treatment costs, reducing the prevalence of physical inactivity has received attention as an important goal [5]. In addition, PA develops as a behavior through complex and dynamic interrelations among individual, social, and environmental factors, underscoring the usefulness of using multidimensional models to study PA [6,7]. As an example of this approach, the socio-ecological model of McLeroy et al. [8] was used to study the barriers and motivations to PA in the elderly. The aim of this study was to systematically review the motivators and barriers to PA in people aged 60 years and older in Iran and other countries.

## MATERIALS AND METHODS

### Search strategy

**Figure 1.**
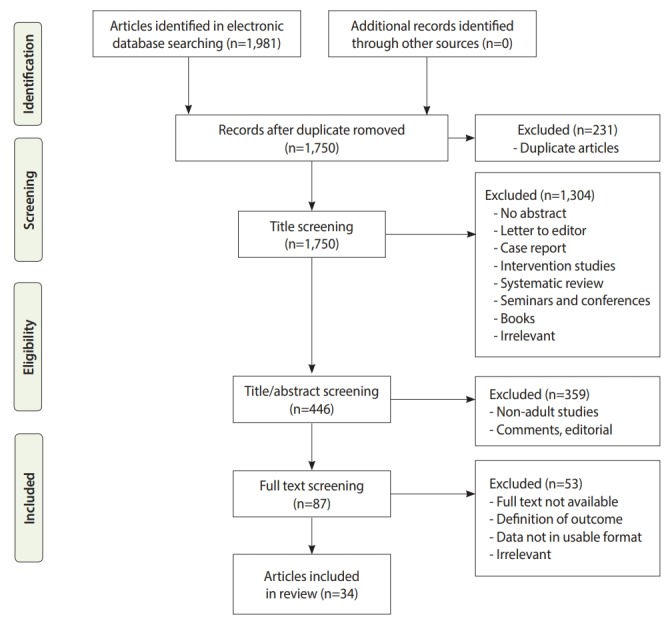
The literature review and retrieval process.

To collect the data, a comprehensive search was performed of several electronic databases (PubMed [MeSH terms], Web of Science, Scopus, Embase, Scientific Information Database, Magiran) to identify all potentially relevant publications in the Persian and English languages from 2000 to November 2017. The following keywords were used: “aged (or age 60 and over),” “physical activity (or exercise),” and “motivator and barrier” (Supplementary Material 1). The detailed search strategy implementation in PubMed was as follows: ([elderly OR Aged OR “60 over aged”] AND [“Physical activity” OR “Exercise” OR “Physical exercise” OR “Motor activity”] AND [Motivation OR Motivat* OR Barrier*]). The inclusion criteria were (1) articles published in the Persian and English languages; (2) original research examining the barriers and motivators to PA in the elderly; (3) studies that examined outcomes in terms of physical and mental illness among the elderly (aged 60 and over); and (4) studies conducted from from 2000 to November 2017. The exclusion criteria were non-original articles (i.e., letters to the editor, case reports, interventional studies, reviews, meta-analyses, and articles presented at seminars and conferences were excluded), articles with no specific definition of age, those that were conducted before 2000 but were accepted in 2000 or later, articles analyzing elderly individuals living in nursing homes, and articles with a very low quality score. In addition, a backward search (checking bibliographic mining of identified papers for any additional studies) was conducted to identify any studies that were not retrieved using the main search strategy. All quantitative and qualitative designs were included. In total, 1,981 articles were retrieved, of which a total of 34 articles remained after the review process shown in Figure 1. Finally, 2 authors carefully examined 34 full-text articles.

In this study, 2 types of studies were investigated: (1) quantitative studies (5 from Iran and 15 from other countries); (2) qualitative studies (1 from Iran and 13 from other countries).

There were 6 studies in Persian and 28 studies in English.

### Evaluating the quality of articles

The quality of qualitative studies was assessed using the qualitative methodological checklist of the National Institute of Clinical Nursing (NICE) [9]. In general, according to the NICE checklist, ++ means that all or most of the checklist criteria have been fulfilled, + means that some of the checklist criteria have been fulfilled, and – means that few or no checklist criteria have been fulfilled. Quantitative studies were assessed using the Newcastle-Ottawa Quality Assessment Scale (NOS) adapted for cross-sectional studies [10]. The NOS is based on 3 domains, including the selection of study groups, the comparability of groups, and the description of exposures and outcomes. This scale, which includes 8 items is scored in terms of stars, assesses the quality of each study in each domain. All items except the comparability domain have 1 star, while the maximum score for the comparability domain is 2 stars. The total number of earned stars is calculated as the total quality score for each study, which ranges from 1 star (very poor) to 10 stars (high quality). Studies were classified as high-quality (8-10), medium-quality (6-7), or low-quality (<6). Two review authors (SY and HMS) completed the quality assessment independently. In cases of disagreement or items that remained unclear, a third review author (AR or MG) was consulted.

### Data extraction

**Figure 2.**
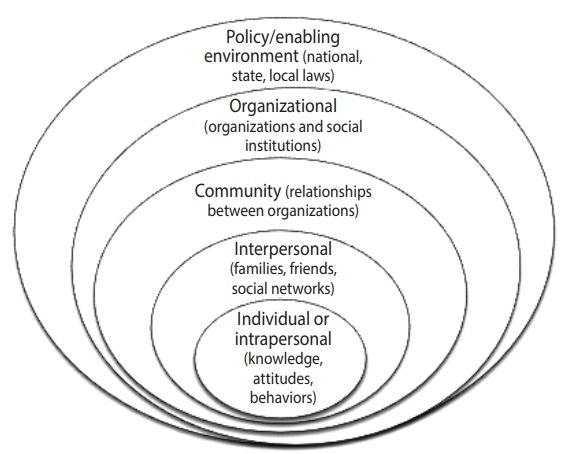
Levels of impact in the socio-ecological model. Adapted from McLeroy et al. Health Educ Q 1988;15:351-377 [8].

We used a structured form to extract the data. The extracted data included study and participant characteristics (e.g., gender, location, country, population, age, type of instrument, type of study, year of study), as well as motivators and barriers to PA. Two authors (SY and HMS), who conducted the study selection independently, performed the data extraction. Any disagreements were discussed with a third review author (AR or MG) if necessary. The data were classified using the socio-ecological model that McLeroy et al. [8] developed in 1988 as a theoretical framework involving interpersonal and intrapersonal factors, organizational and social factors, and environmental factors (Figure 2). The identified factors were prioritized based on the frequency of participants’ responses.

### Ethics statement

This study is a systematic review and does not deal with human participants.

## RESULTS

### Quality of qualitative and quantitative studies

Fortunately, almost all the qualitative studies followed the items in the NICE checklist for qualitative studies. Two of the 14 qualitative articles were high-quality, 12 were good-quality, and 5 were poor-quality, as indicated in Table 1. The quantitative studies were evaluated using the NOS scale, and 18 articles were found to be moderate- to high-quality, while 2 were moderate-quality.

### Description of reviewed studies

The articles were published between 2000 and November 2017, and the sample sizes ranged from 9 (in a qualitative study) to 4,227 participants (in a quantitative study). The samples consisted of both men and women subjects in 30 of the 34 studies, exclusively of women subjects in 4 studies, and exclusively of men subjects in 1 study. The most common type of PA was walking, followed by swimming and fitness, and a description of the type of PA was not available in 17 studies.

In Tables 2 and 3, the characteristics and major findings of the studies are presented.

### Motivators and barriers to the physical activity among the elderly

In the framework of the McLeroy model, we examined the motivators and barriers to PA among the elderly in the intrapersonal, interpersonal, and environmental domains as follows (Tables 2 and 3). According to the study population—people aged 60 years and older—the items from the organizational domain of the McLeroy model were excluded. For quantitative studies, only significant variables were evaluated. Due to the heterogeneity across studies and input variables, it was not possible to conduct a meta-analysis of the results. Below, the most important factors for all dimensions of the model, based on the frequency of participants’ responses, are presented.

#### Intrapersonal factors

Through the literature review, 23 barriers and 16 motivators were identified as intrapersonal factors. In several articles, physical problems—such as difficulty in walking, physical health problems, physical weakness, respiratory problems, and lack of energy—were mentioned as key barriers [13,14,16,19,27,31-34,36, 38-41,43-45]. Time limits were the second most important barrier related to intrapersonal factors [16,17,19,27,28,35,36,39,42-44]. The third most important intrapersonal barrier was fear of falling [14,17,19,23,27,31,35,40,43,45].

The most important intrapersonal set of motivators that resulted in PA was improving one’s physical condition, which included improving one’s balance and walking ability, reducing muscle pain, improving sleep, and strengthening one’s muscles [18,24,34,41,44]. Enjoyment [12,24,40,44], addressing psychological issues (which involved relief from stress, feeling more efficient, having positive perceptions of PA, having a positive self-image, being less depressed, and enhancing sleep [14,16,33,41]), and increasing motivation and access to PA resources [15,30,34,38] comprised the second most important set of intrapersonal motivators [12,24,28,40,44,45]. The third most important set of intrapersonal motivators included a lack of knowledge [17,30], health concerns [17,37], and being economical [24,34].

#### Interpersonal factors

In the interpersonal domain, 6 barriers and 7 motivations were identified (Table 4). The most important interpersonal barrier to PA was having no companion [36,42]. Family responsibilities (taking care of grandchildren, children, and sick people at home) were the second most important interpersonal barrier to PA [34].

The most important interpersonal motivator was being social, which included communication with friends, peer support, communication with others, exercise with friends, social coherence, moderate and high local dependency, an abundance of companions for walking, and support from others [11,16,18,23,24,26,28,30,36,40]. Supervision of health professionals was the second interpersonal motivator [18,28,29]. In addition, 3 articles identified specialist health care [18,28,29] as a motivator for PA. Another important interpersonal motivator for PA was the availability of sports facilities [15,24].

#### Environmental factors

Overall, 7 barriers and 6 motivators were identified at the community level. The most important barrier was physical barriers to walking, which included problems related to safety, parked motorcycles next to the street, potted plants, rubber tiles in playgrounds, food retailers, paved streets, broken sidewalks, scaffolds, snow accumulation along the street in winter, devoted seats in parks for children, the lack of facilities such as benches for resting, poor locations, unsafe roads, stray dogs, and hills [22,23,26,27,31,43,44]. The second most important set of barriers were related to temperature, season, and weather [16,25,27,34,35]. More intense PA among the elderly was observed in the spring (40.1%), in sunny weather (76.8%), and at moderate temperatures (56.2%) [25]. The third most important barrier was a lack of facilities for exercise [16,21,34,36].

The most important environmental motivator was the suitability of the physical environment. This factor included pleasant landscapes, streetlights, sidewalks, bike riding routes, walking paths, the neighborhood’s suitability for walking, interconnections between streets and an attractive environment, an environment free from non-cultural social activities (e.g., smoking, drinking alcohol, gambling), green space, attractive architecture, benches for resting, a place for dog parks, a smooth surface for hiking, and food availability in urban centers [11,23,26,34,36]. Environmental security was the second environmental motivator [26,30].

## DISCUSSION

In the present study, information on PA among the elderly was reviewed from 2000 to November 2017. We aimed to identify the motivators and barriers to PA among individuals aged over 60 in Iran and worldwide; therefore, some studies were excluded because they did not analyze participants under or over 60 years of age as 2 different groups [2,36]. In general, the ecological model is a comprehensive multilevel framework that includes contributors to active behavior at all levels: individual (interpersonal and intrapersonal), social, environmental, and policy [47].

A systematic review by Baert et al. [4] on adults aged over 79 confirmed that quantitative research has a greater focus on the interpersonal and community levels, while qualitative research tends to focus more on the interpersonal level. We found that more research is needed into barriers and motivators at the organizational level, while Baert et al. [4] showed that community-based barriers and motivators need more research because policy-makers may be able to exert influence on these factors.

### Intrapersonal factors

Health status was highlighted in most articles, either as a barrier (18 times) or as a motivator (5 times) for PA. To summarize, in the literature review, 23 articles reported that poor health was a relevant factor for PA among those aged 60 and over. Moreover, the beneficial effects of PA on health status (such as improving balance, improving walking ability, reducing muscle pain, improving sleep, and strengthening) are well established. In addition, Baert et al. [4] reported that health status was both a barrier and motivator. Nonetheless, health improvement has been reported as an important motivator, and research has highlighted that health benefits can be a major factor for promoting PA [17]. In this review, most studies were conducted in Iran and the USA. In these countries, special consideration should be given to the proportion of the young population in light of current barriers. For example, since Iran is a country with a young population, the proportion of the elderly in Iran is expected to peak in the next 50 years, and Iran will face similar challenges to Europe and the USA between 2040 and 2050 [48].

Fear emerged as a special barrier. Fear is a complex phenomenon that can occur in different situations (e.g., fear of walking at night in order to exercise outside the house). It can be related to health status, such as fear of injury or pain, fear of falling, and fear of being dependent on others. Lim & Taylor [49] reported that fear of falling was associated with inadequate levels of PA. Moreover, fear of falling was identified as a barrier to PA in different races, including African-Americans, Whites, and American Indians. In particular, American Indians were worried about falling when there was nobody to help them [50]. Furthermore, anxiety and fear of injury were mentioned as a barrier [19]. Health care providers should recognize this type of fear, and should consider it as an important barrier to be dealt with appropriately, if necessary.

In our study, time limits or lack of time was identified as a barrier. This barrier has also been described in other studies [4]. In a focus group study, lack of time among people aged over 65 was found to be a barrier to PA [19]. In many countries and cultures, the responsibilities of taking care of children and the home take up many hours in the day, with consequent negative effects on health behavior [51].

Enjoying PA was reported as a motivator, as in other studies. Factors related to enjoyment increase the pleasure experienced during PA, which depends on individual preferences (doing physical exercise in a group or enjoying the landscape). Fortunately, health care providers can provide support and guidance in this respect [4].

### Interpersonal factors

As has been found for other age groups, being social was identified as a motivator. In general, social support was also reported as a motivational factor. However, elderly individuals need more social support than younger adults [4]. Unfortunately, elderly people are often single, causing them to be socially isolated [24]. Supervision by health professionals has an important impact on PA in the elderly; in particular, health care providers can encourage elderly people to participate in group exercises. It has been suggested that by providing information and raising awareness about PA, health care providers can augment the self-confidence of elderly individuals to begin exercise regimens [18].

### Environmental factors

A lack of sports facilities was considered to be an especially important barrier. In this regard, construction of playgrounds, sidewalks, parks, or other fitness facilities could motivate individuals to participate in exercise, such as walking. Governments play an important role in providing subsidies and funding for health facilities, such as health centers and walking paths [2]. It was found that a lack of adequate facilities in organizational settings led to a decrease in enthusiasm for PA [52]. Likewise, in Iran, a study confirmed that the presence of exercise facilities, parks, and walking or cycling routes increased elderly individuals’ motivation to engage in PA [16].

Some studies have shown that the availability of a resting place, such as benches along walking paths, may facilitate walking among the elderly. In this regard, for the elderly, it is very important to ensure easy access to safe, beautiful, and interesting places for walking. For these reasons, the elderly were found to prefer routes with places for them to rest [25].

The weather, season, and temperature were identified as potential barriers. Nadri et al. [16], in Iran, reported that participants considered an inappropriate environment to be a barrier. In addition, other studies have shown relationships between natural changes (season, weather, and temperature) and the intensity of PA [25]. In general, more intense PA was observed in the spring (40.1%), in sunny weather (76.8%), and at moderate temperatures (56.2%). Elderly individuals were found to engage in more frequent walking in sunny weather than in rainy weather, and their walking rate was higher at temperatures below 60°F than at high temperatures (81°F). Schmidt et al. [53] reported that “unpleasant weather” such as cold, snow, and extreme heat was a barrier. In addition, a study confirmed that the weather was a potential barrier for the oldest old people [4].

It is worth mentioning that we found some similar barriers and motivators. In some reviewed studies, a link was found between the benefits of prayer and PA. The energy cost of Muslim daily prayers was about 80 calories per day, implying that daily prayers could be considered a form of PA [12,54].

There are two important implications of these findings. First, the factors identified as important herein should be analyzed with regard to gender. Second, it is necessary to consider geographical areas and the accessibility of facilities. Additionally, the type of intervention program (community- or individual-based) is an especially important factor for encouraging the elderly to participate in exercise.

This systematic review has the following limitations. First, no information was extracted on differences between men and women. Therefore, further research is needed to identify any such potential differences. Second, it is possible that differences existed among participants and authors in the definition of exercise and PA. Third, most studies were conducted in developed countries, which may have yielded country-specific results and decreased the generalizability of the findings. As a final limitation, due to inconsistencies and heterogeneity in the data gathering and analysis methods used in the quantitative articles, it was not feasible to conduct a meta-analysis.

## CONCLUSION

This study presents a comprehensive literature review and analysis on the barriers and motivators to PA in the elderly. Most barriers involved the intrapersonal and interpersonal domains. According to the population composition of the countries that were analyzed—especially Iran—interventions to address this issue are essential. In addition, the elderly may have different perceptions of barriers and motivators that need to be considered.

## Figures and Tables

**Table 1.** Quality assessment of qualitative articles

Study	1.1	1.2	2.1	3.1	4.1	4.2	5.1	5.2	5.3	5.4	6.1	6.2
Yoo et al. 2017 [11]	+	+	+	+	+	+	+	+	+	+	?	?
Horne et al. 2012 [12]	+	+	+	+	+	+	+	+	+	+	+	?
Welmer et al. 2012 [13]	+	+	+	+	-	+	+	+	+	+	+	?
Simmonds et al. 2016 [14]	+	+	+	+	+	+	+	+	+	+	+	?
Bethancourt et al. 2014 [15]	+	+	+	+	+	+	+	+	+	+	+	+
Nadri et al. 2016 [16]	+	+	+	+	+	+	+	+	+	+	+	+
Costello et al. 2011 [17]	+	+	+	+	+	+	?	+	+	+	?	?
De Groot et al. 2011 [18]	+	+	+	+	+	+	?	+	+	+	+	+
Lees et al. 2005 [19]	+	?	+	+	+	+	?	+	+	+	?	?
Grossman et al. 2003 [20]	+	+	?	+	?	+	+	?	+	+	?	-
Chastin et al. 2014 [21]	+	+	?	+	+	+	+	+	+	+	+	?
Chen et al. 2015 [22]	+	+	+	+	+	+	+	?	+	+	?	?
Chippendale et al. 2015 [23]	+	+	?	+	?	+	+	+	+	?	+	+
Miller et al. 2017 [24]	+	+	+	+	+	+	+	+	+	+	?	?

1.1, whether the qualitative approach is appropriate; 1.2, whether it is clear what the study is doing; 2.1, how defendable is the research design/research methodology?; 3.1, how were the data collected?; 4.1, whether the text was clearly explained; 4.2, whether the methods were reliable; 5.1, whether the data were rich; 5.2, whether the analysis was reliable; 5.3, whether the findings are persuasive; 5.4, whether the conclusion is sufficient; 6.1, whether the study was approved by an ethics committee; 6.2, whether the role of the researcher was clearly explained; +, appropriate/clear/defendable/persuasive/rich/yes; -, inappropriate/not defendable/not clear/not persuasive/not/poor; ?, I’m not sure/combined/reported.

**Table 2.** Description of the findings reported in eligible studies

Study		Place of research	Size of samples with gender separation	Age (yr)	Race	Location	Type of instrument	Type of physical activity	Socio-ecological model^1^			
									Intrapersonal	Interpersonal	Environmental	Organizational
Cross-sectional study												
	Price et al., 2012 [25]	Spartanburg, South Carolina	488 women, 565 men	>60	Often White	Not stated	Observation	Walking	-	-	o	-
	Van Holle et al., 2015 [26]	Belgium	433; 54% women	>65	Not stated	Residential house	Questionnaire	Walking	o	o	o	-
	Eronen et al., 2014 [27]	Finland	848; 53% women	75-90	Not stated	Residential house	Questionnaire	Walking, gardening	o	o	o	-
	Patel et al. 2013 [28]	Auckland, New Zealand	32 men, 48 women	65-75	Not stated	Residential house	Questionnaire	Not stated	o	o	-	-
	Cohen-Mansfield et al., 2004 [29]	Not stated	324; 58% women	74-85	Not stated	Residential house	Questionnaire and telephone interviews	Walking and exercising in class	-	o	o	-
	Yi et al., 2016 [30]	Shandong, China	580 men, 1,000 women	60-85	Not stated	Local community	Questionnaire	Not stated	o	o	o	-
	Rantakokko et al., 2010 [31]	Finland	1,310 people; 75% women	75-81	Not stated	Community	Face-to-face interviews	Walking	o	-	o	-
	Rowiński et al., 2017 [32]	Poland	1,004 men, 1,649 women	>65	Not stated	Not stated	Questionnaire	Walking, biking, swimming	o	o	-	-
	Bird et al., 2009 [33]	Australia	197 women, 136 men	>60	Anglo, Croatian, Greek, Italian, Messi, Maltese, Vietnam	Residential house	Questionnaire	Gymnastics, aerobics, cycling, dancing, horticulture, walking	o	-	o	-
	Macniven et al., 2014 [34]	South Wales	769 men, 1,053 women	≥65	Not stated	Residential house	Interviews	Walking, biking, swimming, dancing, gardening, yoga, fishing, golf, tennis	o	o	o	-
	Bird et al., 2009 [35]	West Melbourne region	72 women	60-84	Italian Vietnamese	Residential house	Questionnaire	Walking, tai chi, yoga, swimming, dancing, gardening, aerobics, house chores	o	o	o	-
					Anglo-Celtic							
	Kowal et al., 2007 [36]	North America	149 women, 21 women over 60 years old	>60	Caucasian	Not stated	Questionnaire	Leisure activities, home activity	o	o	o	-
	Newson et al., 2007 [37]	Australia	96 men, 121 women	63-86	Not stated	Residential house	Questionnaire	Fitness	o	-	-	-
	Gillette et al., 2015 [38]	Washington	215 women, 26 men	70	Caucasian	Residential house	Questionnaire	Not stated	o	-	o	-
	Thornton et al., 2017 [39]	USA: Seattle and Baltimore	726 men and women; 53% women	≥66	Non-Spanish and White	Residential house	Online questionnaire and telephone interviews	Walking, running	o	o	o	-
Qualitative study												
	Costello et al., 2011 [17]	America: Montgomery, County, Maryland	31 women	60-94	Not stated	Residential house	Focus group	Treadmills, standing bikes, swimming classes, walking in water, aerobic exercise	o	o	o	-
	de Groot et al., 2011 [18]	Norway	5 men, 5 women	71-91	Not stated	Residential house	Semi-structured interviews, focus group	Walking	o	o	o*	-
	Welmer et al., 2012 [13]	Sweden	6 men, 14 women	80-91	Not stated	Residential house	Focus group	Walking	o	o	-	-
	Yoo et al., 2017 [11]	Seoul, Korea	46 people; 60% women and 39% men	95	Not stated	Residential house	Face-to-face interviews, semi-structured interviews	Walking	-	-	o	-
	Lees et al., 2005 [19]	Rhode Island	57 women, 9 men	>65	Not stated	Not stated	Focus group	Fast walking, swimming, aerobics, dancing, cycling, sports class	o	o	-	-
	Grossman et al., 2003 [20]	California, USA	15 men, 18 women	>75	Not stated	Residential house	Interviews with open questions	Not stated	o	o	o	-
	Simmonds et al., 2016 [14]	Bristol, Southwest England	29 women, 7 men	65-88	Not stated	Residential house	Semi-structured interviews	Walking	o	o	o	-
	Bethancourt et al., 2014 [15]	King County, Washington	24 men, 28 women	66-80	American/Indian, Native Alaska, Asian/African, Black, White, Caucasian	Residential house	Group interviews	Not stated	o	-	o	-
	Horne et al., 2012 [12]	South Asia	16 men, 13 women	60-70	India, Pakistan	Not stated	Focus group, deep interviews	Walking	o	-	o	-
	Chastin et al., 2014 [21]	Glasgow	9 women	>65	Not stated	Residential house	Structured interviews	Not stated	o	o	o	-
	Chen et al., 2015 [22]	Devlin, in the southern part of the city of Tainan in Taiwan	40 men, 60 women	65-90	Not stated	Residential house	Observation, interviews	Walking	-	-	o	-
	Chippendale et al., 2015 [23]	New York City	14 men and women	≥65	Caucasian, Spanish, Black	Not stated	Questionnaire, semi-structured interviews	Not stated	-	-	o	-
	Miller et al., 2017 [24]	Central state in the Midwest	4 men, 6 women	>65	White	Residential house	Semi-structured interviews	Not stated	o	o	o	-

1 Intrapersonal, interpersonal, environmental, or organizational dimension based on the theory.

**Table 3.** Description of the findings reported in eligible Iranian studies

Study		Place of research	Size of samples with gender separation	Age (yr)	Race	Location	Type of instrument	Type of physical activity	Socio-ecological model^1^			
									Intrapersonal	Interpersonal	Environmental	Organizational
Cross-sectional study												
	Nejati et al., 2010 [41]	Tehran	73 men, 80 women	>60	Not stated	Residential house	Questionnaire	Not stated	o	-	-	-
	Khalili et al., 2015 [42]	Kashan	400 - no breakdown by gender	60-90	Not stated	Residential house	Questionnaire	Not stated	o	o	o	-
	Nadri et al., 2016 [16]	Tehran	17 men, 13 women	76-90	Not stated	Residential house	Semi-structured deep interviews	Not stated	o	o	o	-
	Shiraly et al., 2017 [43]	Shiraz	524 men, 476 women	60-80	Not stated	Residential house	Questionnaire	Ordinary walking, fast walking, swimming, gardening	o	o	-	-
	Sharifian et al., 2014 [44]	Kerman	310 - no breakdown by gender	>60	Not stated	Residential house	Questionnaire	Walking, running, ball sports, cycling, hiking, swimming	o	o	o	-
Qualitative study												
	Salehi et al., 2010 [40]	Tehran	102 men, 298 women	>60	Not stated	Residential house	Questionnaire, structured interviews	Not stated	o	o	-	-

1 Intrapersonal, interpersonal, environmental, or organizational dimension based on the theory.

**Table 4.** Motivations and barriers to physical activity for the elderly

Dimensions	Physical activity	
	Barriers	Motivators
Intrapersonal	Physical problems [13,14,16,19,27,31-34,36,38-41,43-45]	Improving one’s physical condition [18,24,28,34,41,44,45]
	
	Time limits [16,17,19,27,28,35,36,39,42-44]	Enjoyment [12,24,28,40,44,45]
	Fear of falling [14,17,19,23,27,31,35,40,43,45]	Understanding psychological issues [14,16,33,41]
	Fatigue [17,19,21,33,35,42],	Having motivation and access to physical activity resources [15,30,34,38]
	Lack of interest [14,16,32,34,36,44]	Lack of knowledge [17,30]
		Health concerns [17,37]
	Lack of motivation [16,28,32,33,35,43]	Being economical [24,34]
	Pain [21,24,38,41,43]	
		Feeling security [33]
	Laziness [19,36,40,44]	Having a long life [40]
	Financial cost [13,24,32,34]	Fear of falling and illness [13]
		Pain [21]
	Age [42-44]	Loneliness [35]
	Issues related to individual beliefs [13,17]	Socioeconomic status, having sports skills, training [30]
	Household chores [34,36]	Having enough time [12]
	Security concerns [14,23]	Joining physical activity to daily life [14]
	Single and widower status [24,43]	Spending free time [44]
	
	Being active enough [28]	
	Sex [43]	
	
	Lack of energy [21]	
	Insufficient understanding of physical activity [42]	
	An unpleasant experience [18]	
	Lack of self-discipline [17]	
	Low level of education, retirement, life problems [43]	
	Heavy weight [42]	
	Feeling self-awareness [39]	
Interpersonal	Having no companion [36,42]	Being social [11,16,18,23,24,26,28,30,36,40]
	
	Family responsibilities [34]	Specialist health care [18,28,29]
	Having no professional guidance, inadequate information [15]	Availability of facilities [15,24]
	Social pressure, having less time to spend with friends and family [21]	
	Exercise clubs devoted to young adults and the lack of planning in the at clubs [23]	Having a companion for exercise, fear of dependency [13]
	Working with others, different views of others [16]	
		Assessment of exercise program by a professional instructor [29]
	
		Social pressure [21]
Environmental	Physical barriers to walking [22,23,26,27,31,43,44]	Suitability of the physical environment [11,23,26,34,36]
	
	Temperature, season, and weather [16,25,27,34,35]	Environmental security [26,30]
	Lack of facilities for exercise [16,21,34,36]	
		Access to public transportation [11]
	Traffic [16,36]	Access to sports facilities [30]
		Social network of neighbors, air quality, living in an apartment, proximity to sports facilities [23]
	Inappropriateness of the timing of sports classes [16,23]	Economic and financial agents, holding walking meetings [16]
	
	Lack of personal safety [16]	
	Commuting and distance from home to sports facilities [23]	
