# Supplementary material for: A systematic review of barriers and motivators to physical activity in elderly adults in Iran and worldwide
Source: Epidemiol Health. 2019 Nov 29;41:e2019049. doi: 10.4178/epih.e2019049 (PMC6976727; doi:10.4178/epih.e2019049)
Supplement: Supplementary file 1 [file epih-41-e2019049-suppl.docx]

**Supplementary Material 1. Search strategy and keywords**

| **International database** | | |
| --- | --- | --- |
| **Row** | **Database** | **Search strategy** |
| 1 | PubMed | (((Elderly[Title/Abstract] OR Aged[Title/Abstract] OR 60 over aged[Title/Abstract]) AND (Physical activity[Title/Abstract] OR Exercise[Title/Abstract] OR physical Exercise[Title/Abstract] OR Motor activity[Title**/**Abstract]) AND (Motivation[Title/Abstract] OR Motivat*[Title/Abstract] OR Barrier[Title/Abstract]))) |
| 2 | Embase | ('elderly':ab OR 'aged':ab OR '60 over aged':ab) AND ('physical activity':ab OR 'exercise':ab OR 'physical exercise':ab OR 'motor activity':ab) AND ('motivation':ab OR 'motivate':ab OR 'barrier':ab) |
| 3 | Scopus | TITLE-ABS-KEY ( "Elderly" OR "Aged" OR "60 over aged" ) AND TITLE-ABS-KEY ( "Physical activity" OR exercise OR "physical Exercise" OR "Motor activity" ) AND TITLE ( motivation OR "Motivate" OR "Barrier" ) |
| 4 | Web of science | TI=((Elderly OR Aged OR 60 over aged) AND (Physical activity OR Exercise OR physical Exercise OR Motor activity) AND (Motivation OR Motivate OR Barrier))  Timespan: 2000-2017. Indexes: SCI-EXPANDED, SSCI, CPCI-S, CPCI-SSH, ESCI. |
| **Iranian database** | | |
| 1 | Scientific Information Database | Searched in Persian language |
| 2 | Magiran | Searched in Persian language |
